# Supplementary material for: Radiofrequency remote control of thermolysin activity
Source: Sci Rep. 2021 Mar 16;11:6070. doi: 10.1038/s41598-021-85611-w (PMC7971047; doi:10.1038/s41598-021-85611-w)
Supplement: Supplementary file 1 — Supplementary Information. [file 41598_2021_85611_MOESM1_ESM.docx]

Supporting Information: Radiofrequency Remote Control of Thermolysin Activity

*Christian B. Collins*

*Department of Chemistry*

*Campus Delivery 1872*

*Colorado State Unviersity*

*Fort Collins, CO 80523-1827*

[*Christian.B.Collins@gmail.com*](mailto:Christian.B.Collins@gmail.com)

*Ryan A. Riskowski*

*Department of Chemistry*

*Campus Delivery 1872*

*Colorado State Unviersity*

*Fort Collins, CO 80523-1827*

*Present Address:*

*Department of Physics*

*University of Nebraska – Omaha*

*Omaha, NE, 68182*

*rriskowski@unomaha.edu*

*Corresponding Author:*

*Christopher J. Ackerson*

*Department of Chemistry*

*Campus Delivery 1872*

*Colorado State Unviersity*

*Fort Collins, CO 80523-1827*

*ackerson@colostate.edu*

Contents

[Figure S.1 4](#_Toc514689314)

[Synthesis of particles: 4](#_Toc514689315)

[Magnetite Fe_3_O_4_ Core synthesis procedure 4](#_Toc514689316)

[Figure S.2. 4](#_Toc514689317)

[Gold Coating Procedure 5](#_Toc514689318)

[Figure S.3. 5](#_Toc514689319)

[Phase Transfer 6](#_Toc514689320)

[Conjugation 6](#_Toc514689321)

[SDS PAGE of conjugates 7](#_Toc514689322)

[Figure S.4. 7](#_Toc514689323)

[Ratio of thermolysin to particles in conjugation 7](#_Toc514689324)

[Ratio of thermolysin to particles after conjugation and rinsing 8](#_Toc514689325)

[Figure S.5. 8](#_Toc514689326)

[Figure S.6. 9](#_Toc514689327)

[Enzyme assays: 9](#_Toc514689328)

[Thermolysin activity assay and bulk heating experiments 9](#_Toc514689329)

[Figure S.7. 10](#_Toc514689330)

[Figure S.8. 10](#_Toc514689331)

[Figure S.9. 11](#_Toc514689332)

[Figure S.10. 11](#_Toc514689333)

[Radiofrequency heating assays 12](#_Toc514689334)

[Figure S.11. 12](#_Toc514689335)

[Figure S.12. 13](#_Toc514689336)

[Figure S.13. 13](#_Toc514689337)

[Figure S.14. 14](#_Toc514689338)

[Figure S.15. 14](#_Toc514689339)

[Bulk temperature measurements in RF field 14](#_Toc514689340)

[Figure S.16. 15](#_Toc514689341)

[Enzyme Kinetics measurements: 15](#_Toc514689342)

[Figure S.17. 15](#_Toc514689343)

[Equation S.1. 15](#_Toc514689344)

[Figure S.18. 16](#_Toc514689345)

[Figure S.19. 16](#_Toc514689346)

[REFERENCES 20](#_Toc514689352)


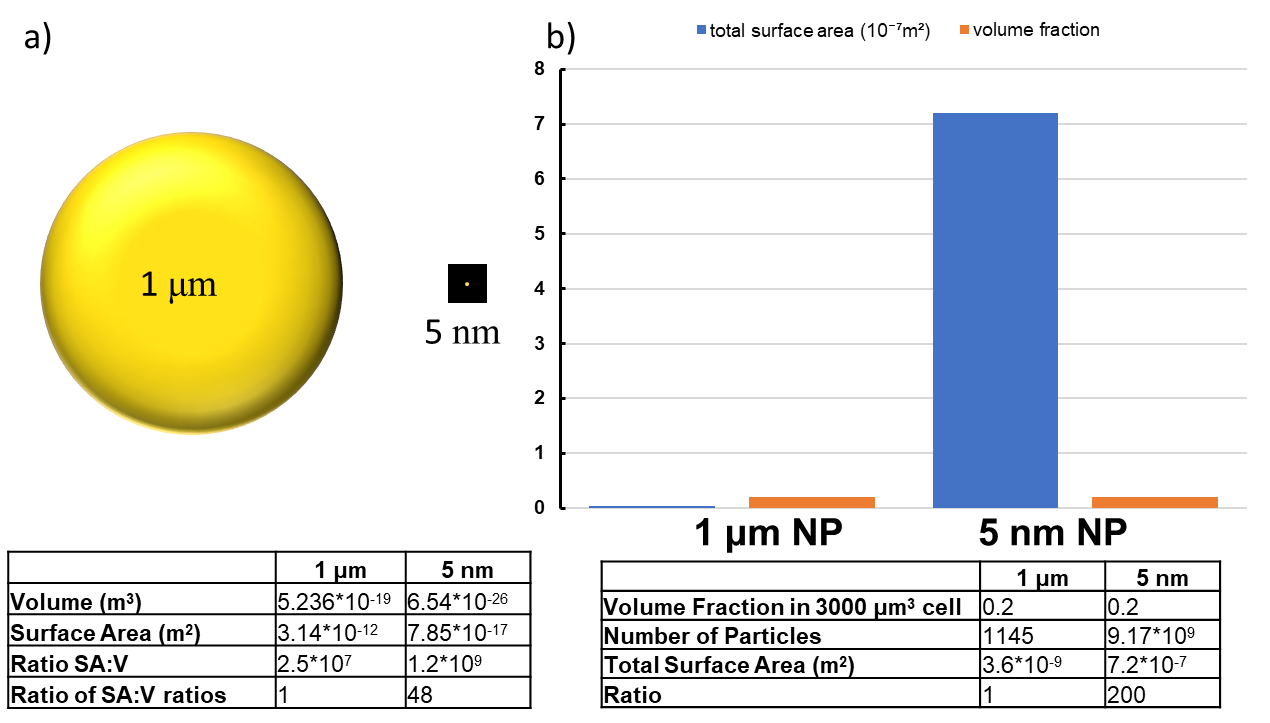
Difference in surface area to volume ratio for 1 micron vs 5 nm particle for fixed volume fraction

Figure S.1**.** a) Comparison of surface area to volume of 1 micrometer and 5 nanometer diameter particles, pictures of particles are to scale. b) Comparison of total surface area (in 10-7 m2 units) from a constant volume fraction of 1 micrometer and 5 nanometer diameter particles.

# Synthesis of particles:

## Magnetite Fe_3_O_4_ Core synthesis procedure

**
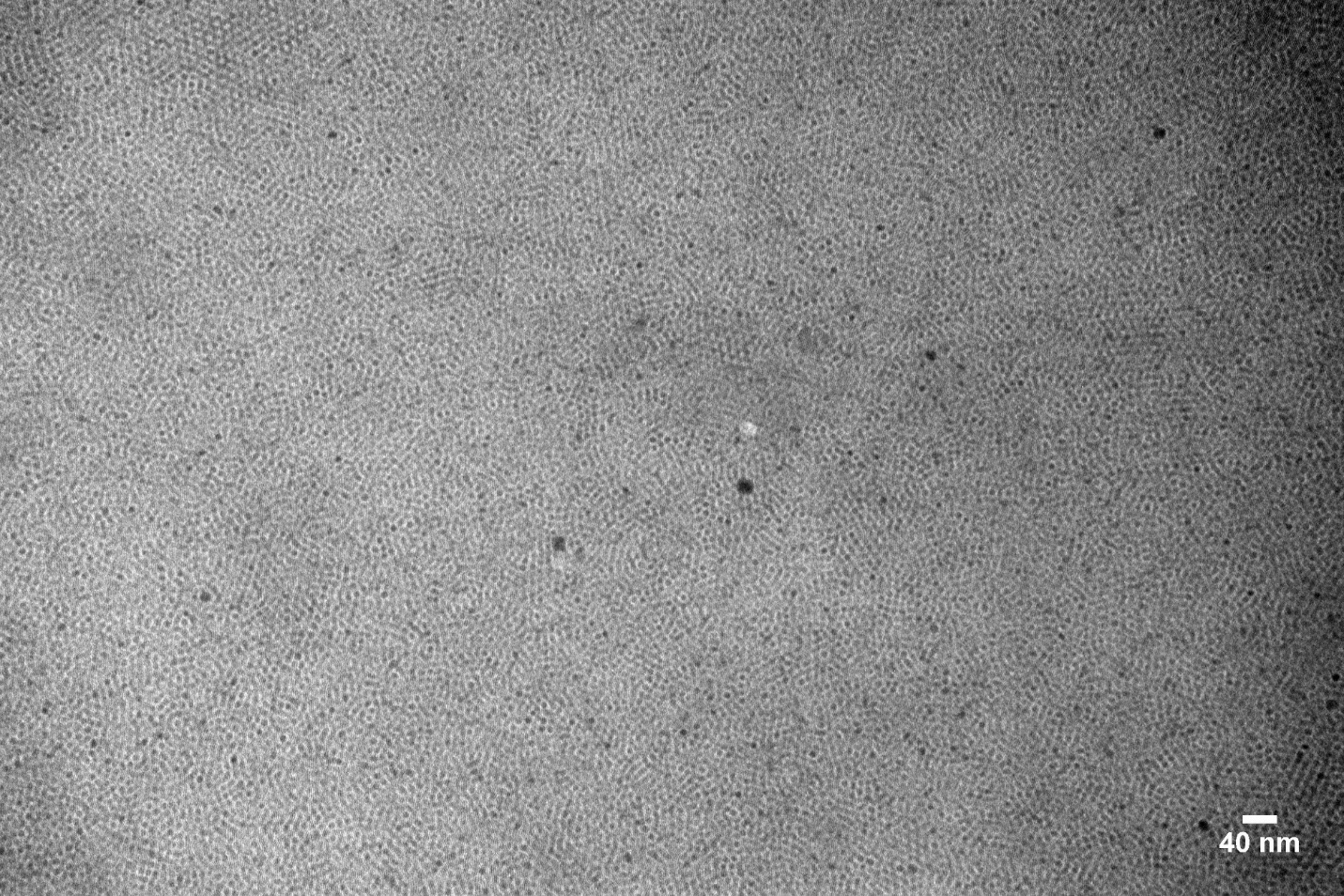
**Magnetite cores were synthesized by thermal decomposition in diphenyl ether following a published method.^1^ Briefly, in a 100 mL 3 neck round bottom flask; 10 mmol 1,2 hexadecane diol and 2 mmol iron (III) acytlacetonate was mixed in 20 mL of diphenyl ether. The solution was then purged and flushed with argon. Then 6 mmol oleylamine (90%) and 6 mmol oleic acid was added via syringe. The solution was then heated to 200^o^C for 30 minutes to boil off impurities. Then it was heated to 265^o^C and refluxed for 1 hour then cooled to 30^o^C, and transferred into storage containers.

Figure S.2. TEM of iron oxide cores, particle size measure by hand in imageJ. Average particle size is 4.5 nm diameter with standard deviation of 0.3 nm. TEM images were taken on a JEOL JEM-1400 Transmission Electron Microscope with an accelerating voltage of 100 kV.

## Gold Coating Procedure

The magnetite cores were coated using another published procedure.^2^ Briefly, 5 mL of the magnetite cores solution from above, without any rinsing or work up was added to a 100 mL, 2 neck, round bottom flask. To that solution, 1.1 mmol gold (III) acetate, 6 mmol of 1,2-hexadecanediol, 0.75 mmol oleic acid and 3 mmol oleylamine was added in 15 mL diphenyl ether. This solution was stirred vigorously at 35^o^C while purging with argon for 20 minutes. Under argon and vigorous stirring the solution was then it was heated to 185^o^C and held at that temperature 1.5 hours. To avoid the freezing of the solvent the solution was then cooled down to 35^o^C and transferred to scintillation vials. The particles were cleaned up by rinsing 3x with 1:1 mixture of hexanes and ethanol. To facilitate faster precipitation a neodymium magnet was placed at the bottom of the scintillation vial. In some cases, it was possible to rinse the particles too much, which could be remedied by adding a small amount of oleylamine back into the solution.


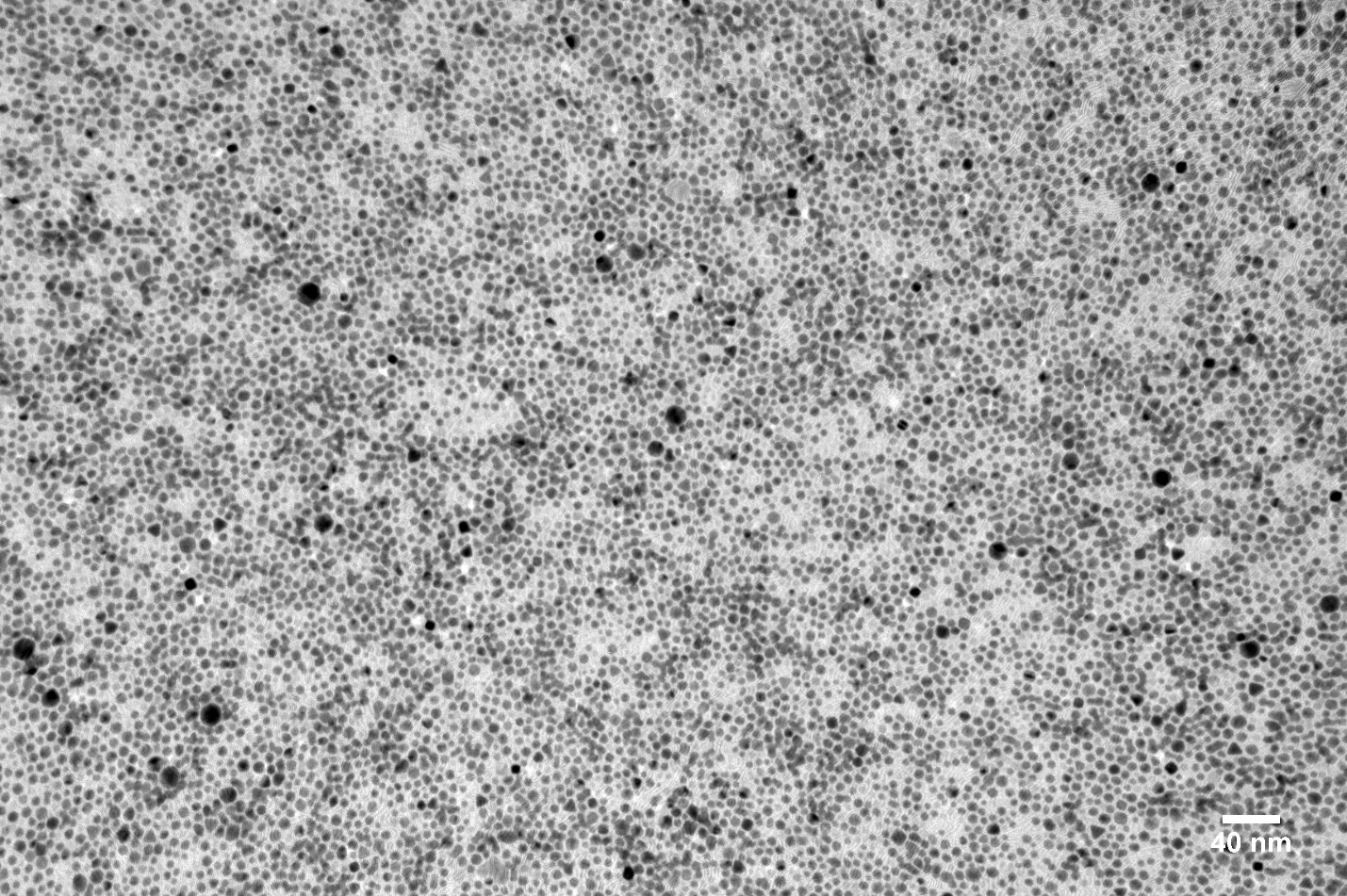


Figure S.3. TEM of gold coated particles, average particle size is 6.1 nm diameter, standard deviation 2.3nm, particle count 4152. Particle size analyzed with imageJ.

## Phase Transfer

To phase transfer the particles a novel two step procedure was used; first an initial exchange and phase transfer from oleylamine coating to 3-mercaptopropanoic acid (3-MPA). Then an exchange of 3-MPA for 11-mercaptoundecanoic acid (11-MUA) to increase solubility in water and biological relevant buffers. In a 20 mL scintillation vial, 2 mL of 17 mg/mL particle solution in heptanes was added with 10 mL of heptanes 5mL of 3-MPA. This is then vortexed overnight. Some phase transfer occurs into the thiol layer almost immediately, but complete exchange was achieved hours later. The particles were then precipitated and washed with 5 mL acetone 3x, until no 3-MPA smell could be detected. The particle pellet was then suspended in 1.5 mL 75 mM tetramethylammonium hydroxide (TMAOH) solution to give a red solution. The alkalinity and the large counter ion (allowing for larger interparticle separation distance) of the TMAOH solution allowed for incredibly stable suspension of the particles.^3^

To improve the stability of the particles in a wide range of solutions the 3-MPA ligands were further exchanged for longer 11-MUA ligands. To the 1.5 mL batch of phase transferred particles, 3.4 mg of 11-MUA was added and the mixture was sonicated for 20 minutes to facilitate dissolution of the 11-MUA. The mixture was then vortexed for 4 hours at room temp. The particles were then centrifugally precipitated using acetone and rinsed 3x with a 1:1 mixture of acetone to 75 mM TMAOH. Then the particles were resuspended in the working buffer for the thermolysin assay, and exhibited a red solution color characteristic of plasmonic gold nanoparticles < 200nm in diameter.

## Conjugation

A novel conjugation procedure was developed to get around the incompatibility of the solubility of the nanoparticles and thermolysin, which is soluble in high ionic strength solutions. To desalt the thermolysin, which is shipped by sigma Aldrich 20% salt by mass because cations are responsible for the remarkable thermal stability of thermolysin, 15 mg was suspended into 3 mL pH 7.5, 50 mM phosphate buffer. This solution was rinsed placed on a rocker for 20 minutes, following by centrifugal precipitation of the enzyme at 4^o^C, 4000 rpm for 20 minutes. Then 10 mg of lyophilized and thoroughly rinsed Fe_3_O_4_@Au NP were dissolved in 3 mL fresh 50 mM pH 6.8 phosphate buffer. The particles were sonicated for ~ 10 minutes until they were clearly in solution. Then 10 mg of HATU was added to the nanoparticle solution and sonicated for 20 minutes. Note the HATU is poorly soluble in water and forms a poor suspension. After sonication, which activates the carboxylic acid groups on the ligands the solution was diluted with 6 mL of pH 6.8, 50 mM phosphate buffer. This was added directly to the washed thermolysin pellet. This was mixed by inversion in hand and then put in a vortexer for 3 hours at 4^o^C. The conjugates were then precipitated via centrifuge, rinsed 2x and then resuspended in 10 mL of pH 7.5, 50 mM phosphate buffer. No enzymatic activity was detected in the second rinsing, indicating very little if any free thermolysin. Besides testing the activity of the conjugates, a SDS PAGE was used to confirm conjugation.

# SDS PAGE of conjugates

A precast BioRad 4-15% gradient gel (product #4561083) was used to confirm a mobility shift in conjugated nanoparticles and thermolysin vs free nanoparticles and thermolysin. Samples were incubated in NewEngland BioLabs purple gel loading dye with SDS (product #B7024S) at 95^o^C for 5 minutes in order to thoroughly denature the enzyme.


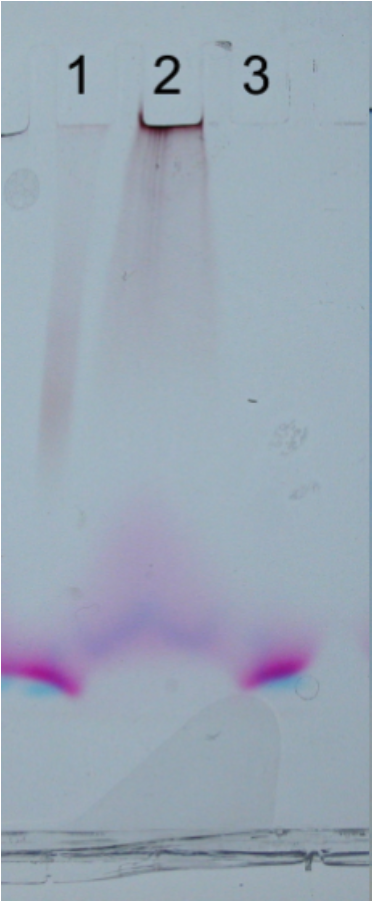

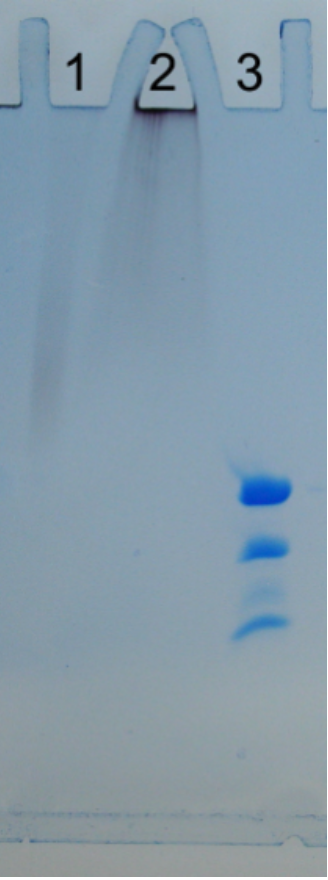


Figure S.4. SDS page gel (biorad precast gradient gel 4-15%) of 1) free nanoparticles (dark red from the plasmon), 2) NP thermolysin conjugates after washing 3) free thermolysin before (left panel) and after (right panel) staining with Coomassie brilliant blue protein stain. The large mobility shift of from the free particles in lane 1 to the conjugated particles in lane 2 confirms the size increase expected with a layer of thermolysin conjugated to it. Note there is also very little to no free thermolysin left over in the conjugates after a thorough rinsing. It is also hard to tell from the blue stain on red particles but the band with reduced mobility in lane 2 exhibits enzymatic activity of thermolysin so it is a strong indicator that there is thermolysin attached to it.

# Ratio of thermolysin to particles in conjugation

Thermolysin from SigmaAldrich is 70% thermolysin by weight, with the rest of the weight being salt, specifically the Ca^2+^ and Zn^2+^ cations necessary for catalytic activity. 15.1 mg was used for the conjugation so there is 10.5 mg of thermolysin. The molecular weight of thermolysin is 34.6 kDa, so the 10.5mg is 3.03*10^-7^ moles or 1.8*10^17^ thermolysin enzymes.

For the particles we used 10mg of 4.5 nm (TEM/core) diameter particles, since the particles are spherical core volume of the particle is So (2.25*10^-7^ cm)^3^ * (4/3) *Pi = 4.8 *10^-20^ cm^3^. From synthesis paper^2^ shell is ~1 nm thick with ratio of Au:Fe weight ratio of 71:29.

For magnetite the unit cell parameter a*b*c=8.39 angst,^4^ so unit cell volume is 5.90*10^-24^ cm^3^, and there are 8 Fe_3_O_4_ in each cell so volume per unit cell 73.8*10^-24^ cm^3^ with 24 Fe and 32 O weight per unit cell is 307.6*10^-21^ g. Weight of iron per unit cell is 2.3*10^-21^ g. From my particle size and unit cell size there are 646 unit cells per particle thus 1.41*10^-16^ g/particle. And weight of Fe per particle is 646*2.3*10^-21^ = 1.48*10^-18^g. So using weight of gold per particle is (1.07*10^-18^g)*(71/29)= 2.62*10^-18^g Au per particle

Surface coverage of 11-MUA 5.70 nm^-2^ which is 5.7 *10^14^ cm^-2^.^5^ Surface area of 6.1 nm particle 4*Pi*(3.05 nm)^2^= 117 nm^2^. Thus, there are 5.7*117= 667 MUA per particle (218.36 g/mol). The weight of 11-MUA 218.36 *6.02*10^23^=3.62*10^-22^ g per 1 MUA ligand. For each particle there are 3.62*10^-22^ *667= 2.4*10^-19^ g MUA so in total 2.4*10^-19^ g MUA + 2.62*10^-18^ g Au + 1.99*10^-16^ g Fe_3_O_4_ = 2.02*10^-16^ g per particle. So, for 10 mg of particles there are have 0.010g /2.02*10^-16^ = 4.9*10^13^ particles. So the ratio I used in conjugation: 1.8*10^17^ thermolysin / 4.9*10^13^ particles = 3670:1 tln:particle.

# Ratio of thermolysin to particles after conjugation and rinsing

Using the molar mass of the particles determined above, a molar absorptivity for the particles was determined using the plasmon absorption peak at 540 nm.


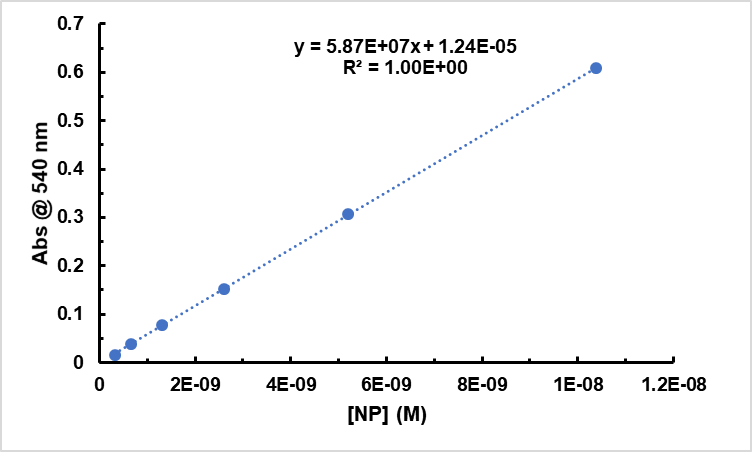


Figure S.5. Standard cure of particle concentration vs UV-vis absorbance at 540 nm

The absorbance from conjugate sample is 0.139 at 540 nm, using the linear fit this corresponds to a concentration of 1.7*10^-9^ M.

The conjugates were then etched with 0.5 M NaCN, for ~15 minutes until red plasmon peak was no longer visible in uv-Vis. Then a Bradford assay was performed to determine the concentration of thermolysin. First, standard solution of known thermolysin concentrations were made, and then reacted with 0.5 mL Bradford reagent (Sigma Aldrich catalog # B6916) for 5 minutes. At that point the uv-VIS absorption was measured at 595 nm. The cyanide etched conjugate sample was then reacted with 0.5 mL Bradford reagent and using the standard curve the absorbance was converted to a concentration of 0.048 mg/mL of thermolysin or 7.9*10^-7^ M. This gives a ratio of 333 mol of TLN:1 mol of nanoparticles.


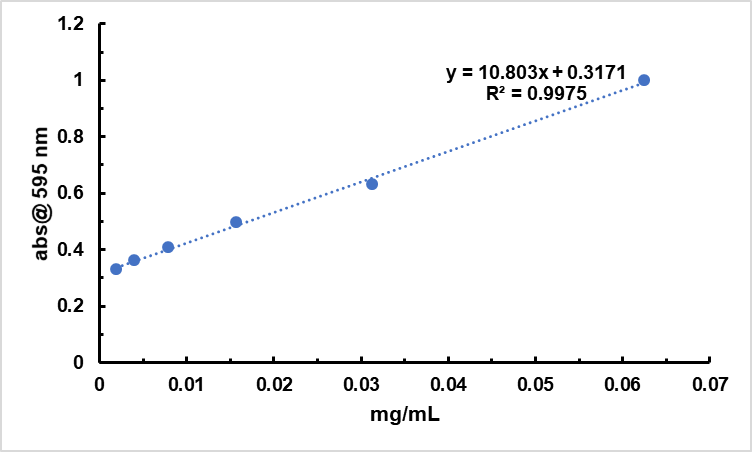


Figure S.6. Bradford assay standard curve of absorbance at 595 nm vs concentration of thermolysin. This was used to determine concentration of thermolysin in conjugates.

# Enzyme assays:

## Thermolysin activity assay and bulk heating experiments

The assay was based on the commercially available Pierce Colorimetric Protease Assay Kit (catalog # 23263) optimized for the specific enzyme conjugates and substrate. Aliquots (for 0,1,2,3 minute time points) of 50 μL of 4 mg/mL succinylated casein in 50 mM pH 7.5 50 mM phosphate buffer were pre-‘heated’ at 17.7^o^C (or different temperature for the temperature studies) in a thermocycler for ~10 minutes along with a buffer blank. Then 5 μL of thermolysin conjugates were added and the samples were heated for 0, 1, 2 and 3 minute time points. To stop the reaction 10 μL of 0.5 M EDTA was added to chelate the catalytic zinc and structural calcium ion, denaturing thermolysin. Then after allowing the reaction to cool back down to room temperature for 5 minutes 25 μL of colorimetric reagent, TNBSA was added. The samples were then incubated at room temperature for 20 minutes, and using the pedestal on a NanoDrop uv-Vis spectrometer the absorbance was measured at 430 nm to monitor formation of the orange product that forms when primary amines react with TNBSA. The slope of the line was then used to calculate the velocity of the enzyme
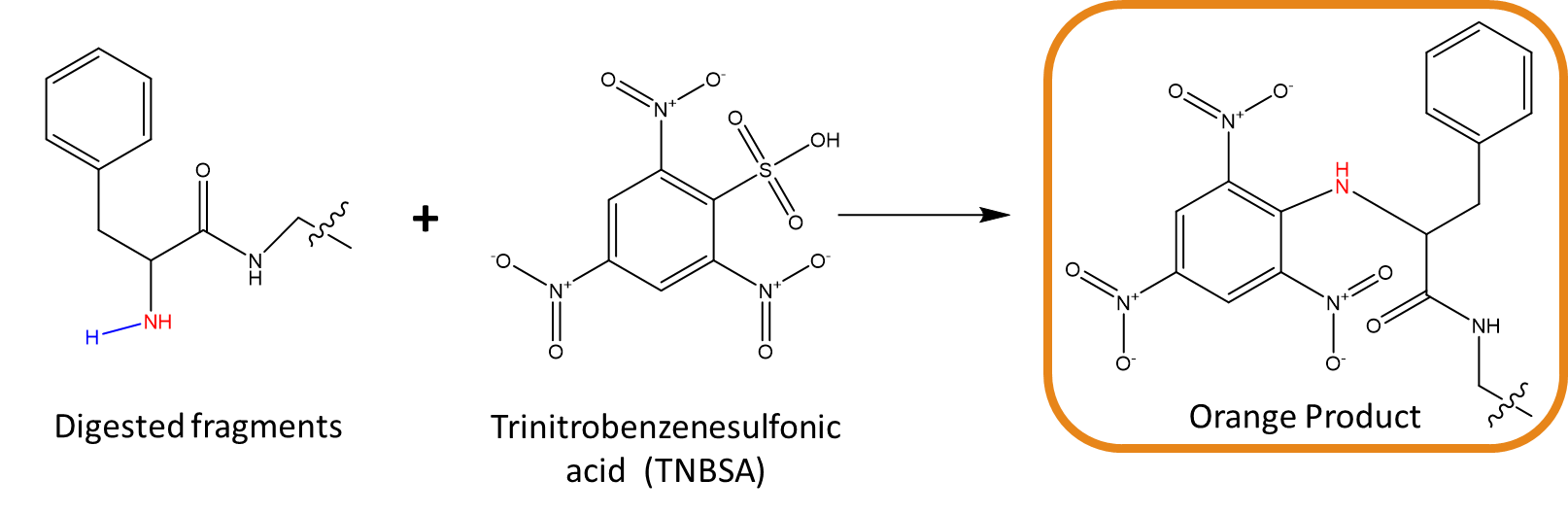
reaction.


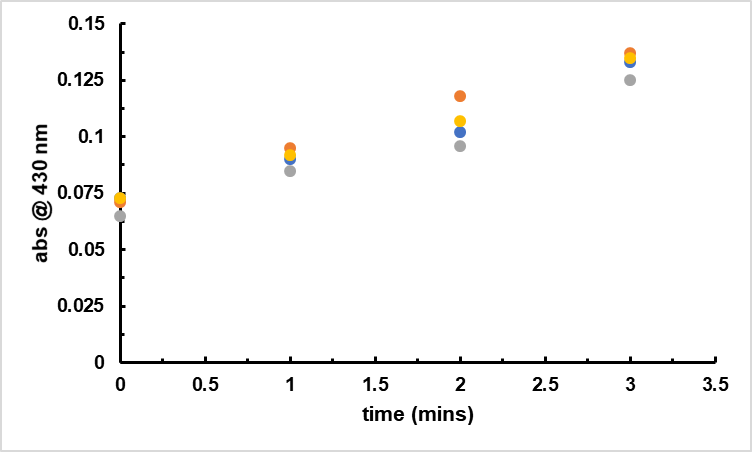
Figure S.7. Reaction scheme for TNBSA with digested fragments

Figure S.8. Bulk heating in absence of RF field at 17.7^o^C, average slope of 4 trails (activity) was 0.02 with a standard deviation of 0.0014.

**
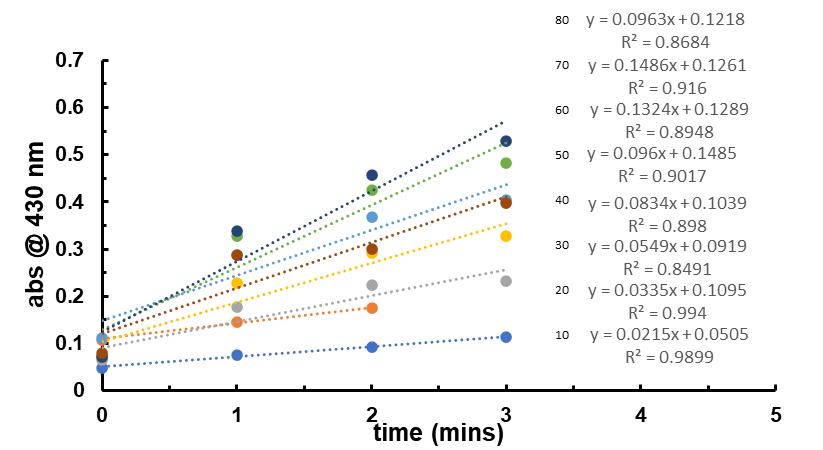
**Figure S.9. Assay results for temperature study. Bulk heating conjugates at 10^o^C (blue), 20^o^C (orange), 30^o^C (grey), 40^o^C (yellow), 50^o^C (maroon), 60^o^C (green), 70^o^C (dark blue), 80^o^C (light blue) .

Figure S.10. Summary of slopes obtained from bulk heating experiments in figure S.8.

# Radiofrequency heating assays


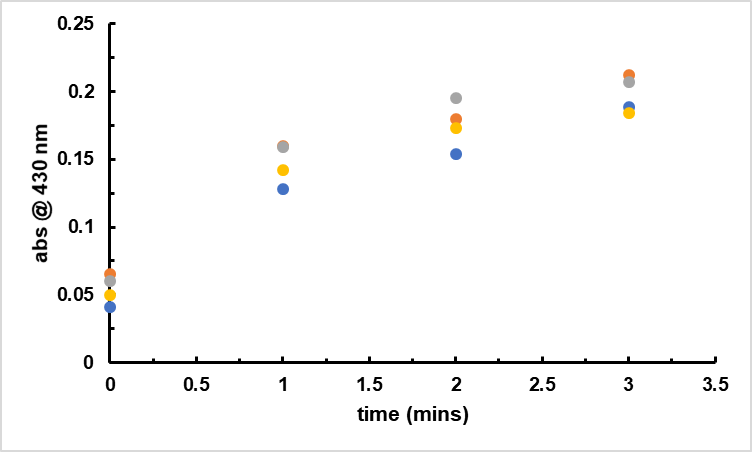
 The exact same assay procedure was used for the RF heating experiments so that they were comparable. The fields were generated using a homemade water-cooled solenoid, with 14 turns and a length of 16 cm. We measured the field strength with a hall effect probe and found it was 0.021T in the center where the samples were placed. It was powered by a Philips PM5192 function generator to input a 17.76 MHz sinusoidal signal into a IFI scx100 power amplifier. An impedance matching box was used to tune the reflected power to zero, new tunings were required for changes in frequency and applied power. The bulk solution temperature was monitored with a Neoptix Nomad fiber optic temperature probe and then the conjugates were pre- heated in a thermocycler as described above to the same temperature, and the velocities of the two different heating methods were compared. Temperature measurements were made consistent by using a stand that holds the fiber optic probe in the same location for every sample.

Figure S.11. RF Heating at 80W, average slope of 4 trials (activity) is 0.046 with a standard deviation of 0.0019


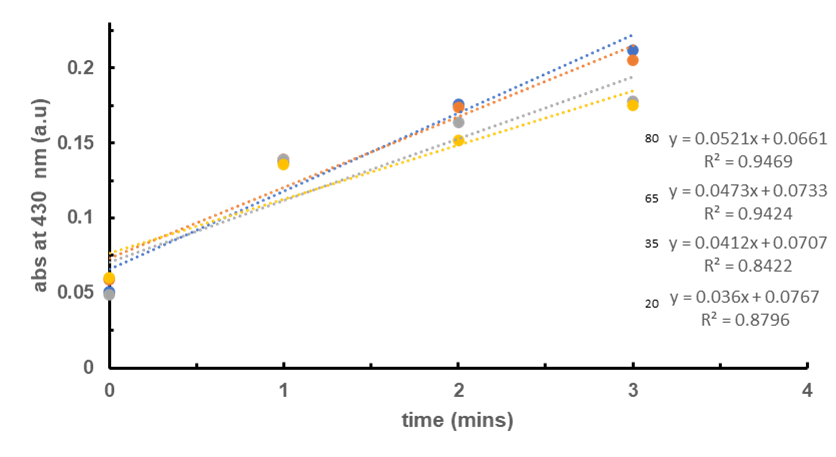

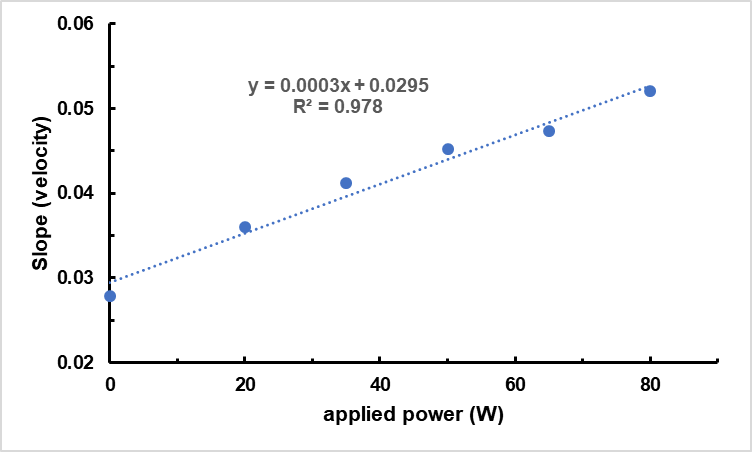
Figure S.12. Activity assay of RF power study to determine activity as a function of power. Here yellow is 20W with a slope of 0.036, grey is 35W with a slope of 0.041, orange is 65W with a slope of 0.047, blue is 80W with a slope of 0.052.

Figure S.13. Plot of activity in RF vs applied power from the data in figure above. Velocity units are (min^-1^). On the right is the linear fit used to make figure 3 in the paper.


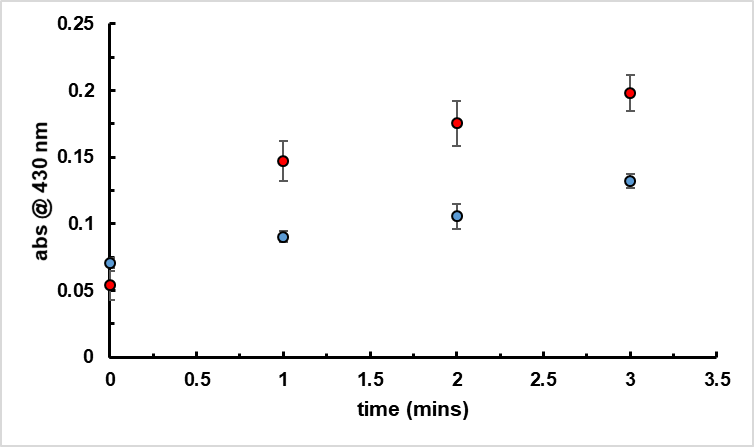
Figure S.14. Combined bulk at 17.7^o^C vs RF heating at same temperature but in an 80W RF field. Error bars represent standard deviation of measurements in quadruplicate.

# Bulk temperature measurements in RF field

To determine the temperature to compare RF heating trials to bulk heating trials the bulk solution temperature of the reaction mixture had to be measured. This is not something that can be done with a normal thermometer, due to the small volume sizes (0.5 mL) or a thermocouple, because the thermocouple interacts with the field and heats itself. Instead we must use a fiberoptic temperature probe, in this case it’s a Neoptix Nomad fiber optic temperature probe.

**
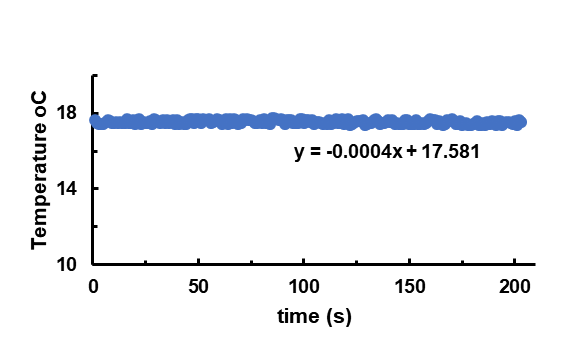
**

Figure S.15. Temperature measurement of 80 W RF sample over 3 minutes. The water cooling system kept the samples at essentially the same temperature for all runs. The 80 W heated the coil the most so its temperature was used for the temperature of all the bulk heating experiments (17.7^o^C).

# Enzyme Kinetics measurements:


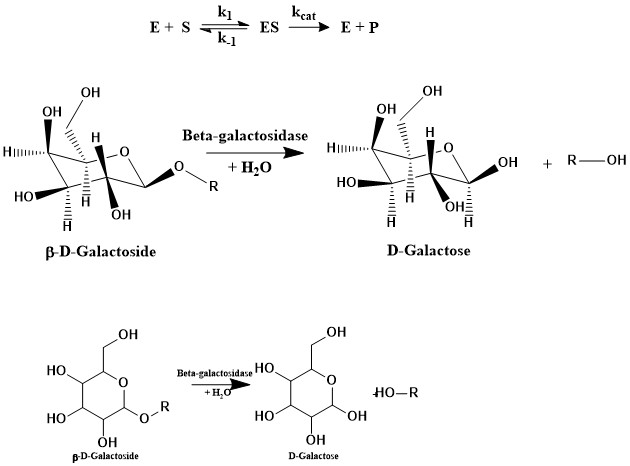
The same enzyme assay was used for kinetics experiments, but the substrate concentration was varied so that the Michaelis Menten kinetics model could be used to investigate differences in RF fields vs the absence of RF fields. The assumption for this kinetics model is that the catalysis happens in two distinct steps, substrate binding and catalysis.

Scheme S.1. Simplified reaction scheme for enzyme catalysis used in Michaelis Menten enzyme kinetics model.

Equation S.1. Linear fit equation to find kinetic parameters V_max_ (maximum velocity when saturated with substrate) and K_m_ (Michaelis constatnt). From this information k_cat_ can be calculated.

Equation S.2. Equation for definition of K_m_.

Equation S.3. Equation to find k_cat_ from V_max_.


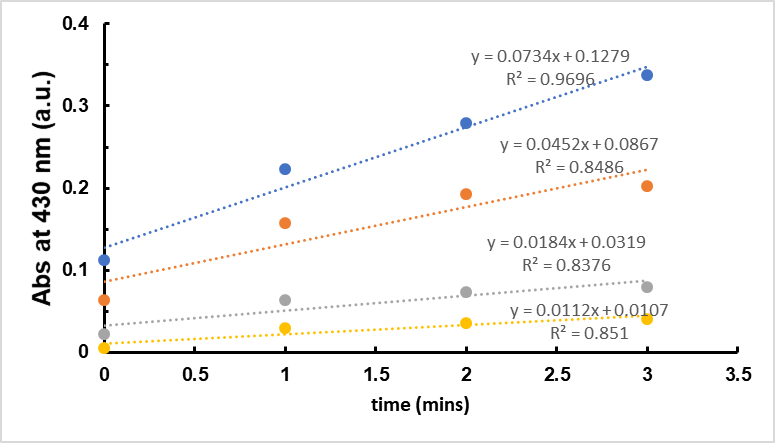

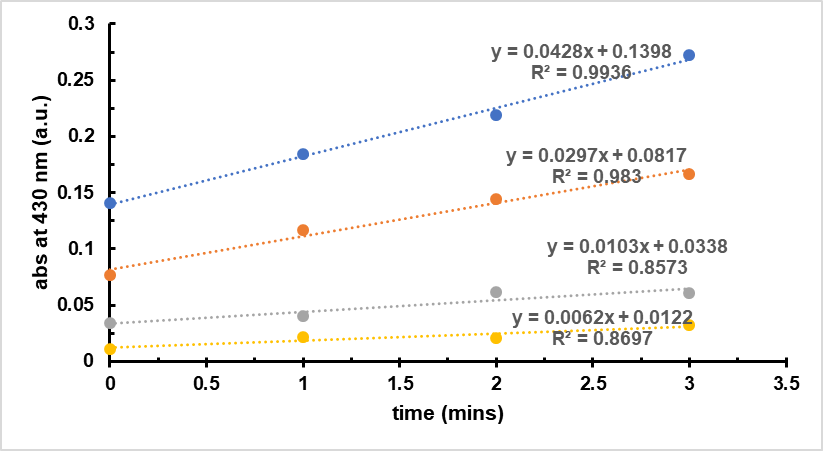
Figure S.16. Bulk ‘heating’ at 17*C absorbance measurements of for activity of thermolysin at substrate concentrations of 0.2 (yellow), 0.5 (grey), 2 (orange) and 4 mg/mL (blue).

Figure S.17. RF at 50W absorbance measurements of for activity of thermolysin at substrate concentrations of 0.2 (yellow), 0.5 (grey), 2 (orange) and 4 mg/mL (blue)

Once the velocities were found, from the slopes of the lines shown in the figures above, these were converted from units of min-1 to M product produced per min using the molar absorptivity of TNBSA with a primary amine.^6^


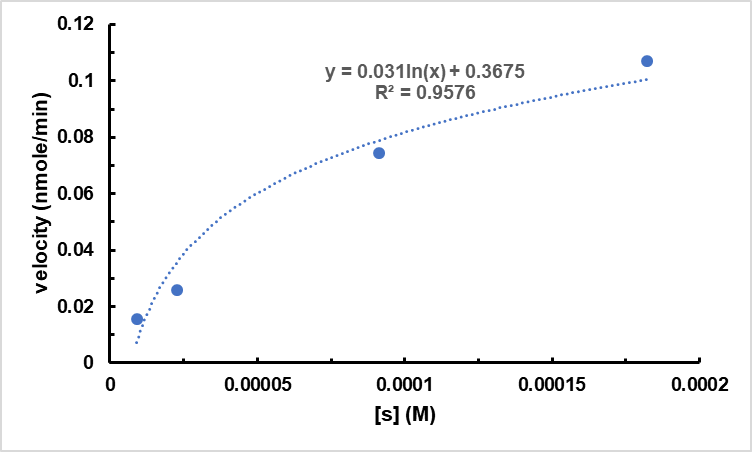


Figure S.18. Results of velocity vs substrate concentration for bulk heating experiments at 17^o^C.


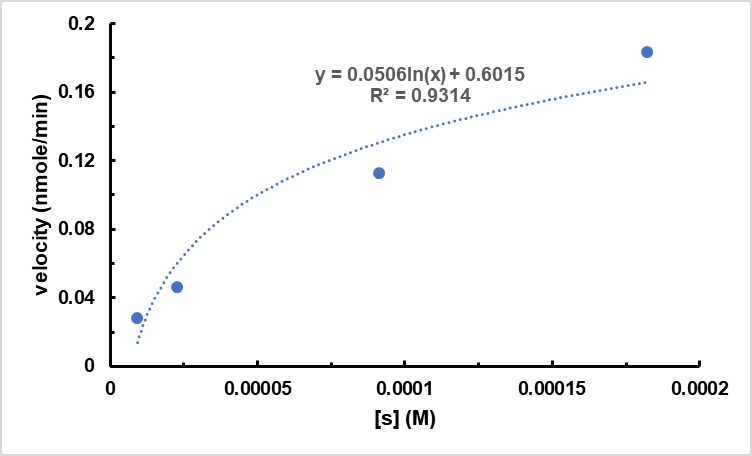
Figure S.19. Results of velocity vs substrate concentration for RF heating experiments at 50 W.

# REFERENCES

1. Sun, S.; Zeng, H.; Robinson, D. B.; Raoux, S.; Rice, P. M.; Wang, S. X.; Li, G. Monodisperse MFe2O4 (M = Fe, Co, Mn) Nanoparticles. *J. Am. Chem. Soc.* **2004**, *126*, 273–279.
2. WangWang; Luo, J.; Fan, Q.; Suzuki, M.; Suzuki, I. S.; Engelhard, M. H.; Lin, Y.; Kim, N.; Wang, J. Q.; Zhong, C.-J. Monodispersed Core−Shell Fe3O4@Au Nanoparticles. *J. Phys. Chem. B* **2005**, *109*, 21593–21601.
3. Laaksonen, T.; Ahonen, P.; Johans, C.; Kontturi, K. Stability and Electrostatics of Mercaptoundecanoic Acid-Capped Gold Nanoparticles with Varying Counterion Size. *ChemPhysChem* **2006**, *7*, 2143–2149.
4. Kokate, M.; Garadkar, K.; Gole, A. One Pot Synthesis of Magnetite–silica Nanocomposites: Applications as Tags, Entrapment Matrix and in Water Purification. *J. Mater. Chem. A* **2013**, *1*, 2022–2029.
5. Hinterwirth, H.; Kappel, S.; Waitz, T.; Prohaska, T.; Lindner, W.; Lämmerhofer, M. Quantifying Thiol Ligand Density of Self-Assembled Monolayers on Gold Nanoparticles by Inductively Coupled Plasma–Mass Spectrometry. *ACS Nano* **2013**, *7*, 1129–1136.
6. Hatakeyama, T.; Kohzaki, H.; Yamasaki, N. A Microassay for Proteases Using Succinylcasein as a Substrate. *Anal. Biochem.* **1992**, *204*, 181–184.
